# When One Fracture Hides Another: A Case Highlighting Cognitive Bias in Trauma Reporting

**DOI:** 10.7759/cureus.97715

**Published:** 2025-11-24

**Authors:** Abdul Rehman, Mujtaba Javed, Raja Ansar Hameed, Atta ur Rahman

**Affiliations:** 1 Emergency Department, St. Luke's General Hospital, Kilkenny, IRL

**Keywords:** anchoring bias, atlanto-occipital subluxation, c1 lateral mass fracture, cervical spine trauma, cognitive bias, missed diagnosis, occipital condyle fracture, radiologic review, satisfaction-of-search, spine imaging

## Abstract

Fractures of the occipital condyle and the C1 lateral mass are rare and often difficult to detect on imaging, particularly when other, more obvious spinal injuries are present. We describe a 48-year-old male who sustained an unwitnessed fall, resulting in severe neck pain. Initial CT scan, follow-up MRI, and X-rays revealed only a fracture of the C7 spinous process. The patient was managed and discharged with cervical immobilization in a Miami J collar, but ongoing symptoms persisted. Only upon retrospective review by a different radiologist, after a repeated presentation to the emergency department and incidentally while interpreting unrelated shoulder imaging, were previously missed injuries detected: a right occipital condyle fracture, a right C1 lateral mass fracture extending into the foramen transversarium, and atlanto-occipital subluxation.

Multiple cognitive biases, including anchoring, satisfaction-of-search, and adherence bias, influenced the delayed diagnosis. The patient was referred to a higher-level trauma center but did not attend follow-up appointments and was lost to follow-up. This case emphasizes the importance of systematic imaging review, awareness of cognitive biases, and correlation of imaging findings with persistent clinical symptoms to prevent missed diagnoses in cervical spine trauma.

## Introduction

Injuries to the occipital condyles and C1 lateral masses are uncommon but clinically significant, with an incidence of about 1-3% in blunt trauma, and up to 4-16% when modern CT imaging is routinely used. C1 (atlas) fractures make up roughly 2% of all spinal fractures and about 10-13% of cervical spine fractures, with lateral mass involvement reported in approximately 25-50% of C1 fracture cases [1,2]. Missed diagnoses can result in craniocervical instability, permanent neurologic deficits, and vascular complications [1,2]. CT and MRI are essential tools for evaluating these injuries; however, subtle fractures and subluxations can easily be overlooked, particularly in the presence of more apparent injuries to the lower cervical spine [3]. Cognitive biases in radiology, such as anchoring, satisfaction-of-search, and adherence bias, can further contribute to missed diagnoses [4,5]. Anchoring occurs when initial findings unduly influence subsequent interpretation. Satisfaction of search arises when the detection of one abnormality leads to the premature termination of the image review [4,5]. Adherence bias is the tendency to rely heavily on prior reports, potentially ignoring persistent or evolving clinical concerns [6].

We report a case in which multiple upper cervical injuries were initially missed due to these cognitive biases, highlighting the importance of systematic review of the craniovertebral junction, careful assessment of subtle injuries, and re-evaluation of prior imaging when patients continue to experience symptoms. This case emphasizes the critical role radiologists play in early recognition and prevention of complications in cervical spine trauma.

## Case presentation

A 48-year-old male presented to the emergency department following an unwitnessed fall on a staircase, resulting in a head injury and transient loss of consciousness. His past medical history included depression, alcohol, opioid, and diazepam misuse. He reported headache and severe neck pain on presentation but denied nausea, vomiting, seizures, or other neurological symptoms. On examination, he was alert, with a Glasgow Coma Scale of 15/15, and hemodynamically stable. Palpation revealed tenderness along the entire cervical spine, but no neurological deficits were observed. Spinal precautions were implemented immediately, and an urgent computed tomography (CT) scan of the brain and cervical spine was ordered.

**Figure 1:**
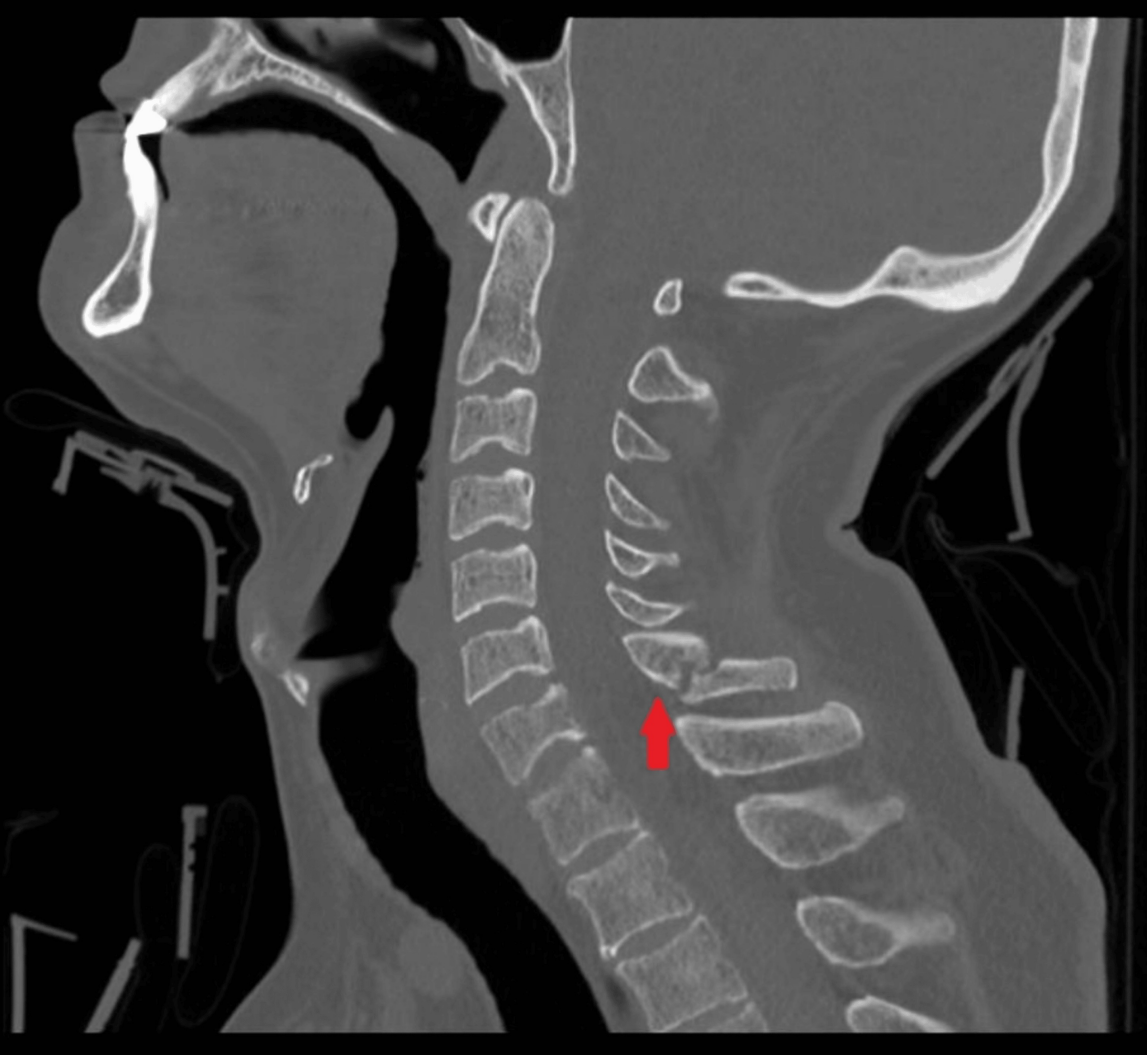
Sagittal CT image of the cervical spine demonstrating an oblique fracture (red arrow) through the mid to proximal portion of the C7 spinous process. The fracture line is clearly visualized extending obliquely through the spinous process, with minimal displacement and preserved alignment of the adjacent vertebral bodies. CT: computed tomography

The CT scan of the brain was normal, without any intracranial bleeding or skull fracture. Cervical spine CT was reported as an oblique fracture through the mid-proximal C7 spinous process without involvement of the lamina, pedicles, or facet joints (Figure 1). No subluxation, dislocation, or other cervical spine abnormalities were reported. The patient was admitted under the spine surgery team and fitted with a Miami J collar.

**Figure 2:**
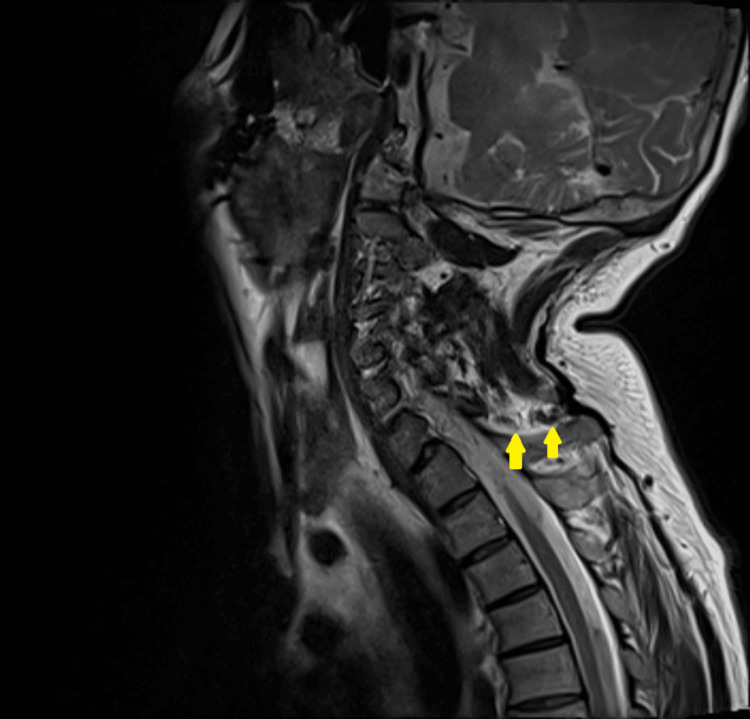
Sagittal MRI of the cervical spine demonstrating an interspinous ligament tear and surrounding edema immediately below the C7 spinous process fracture (yellow arrows). MRI: magnetic resonance imaging

**Figure 3:**
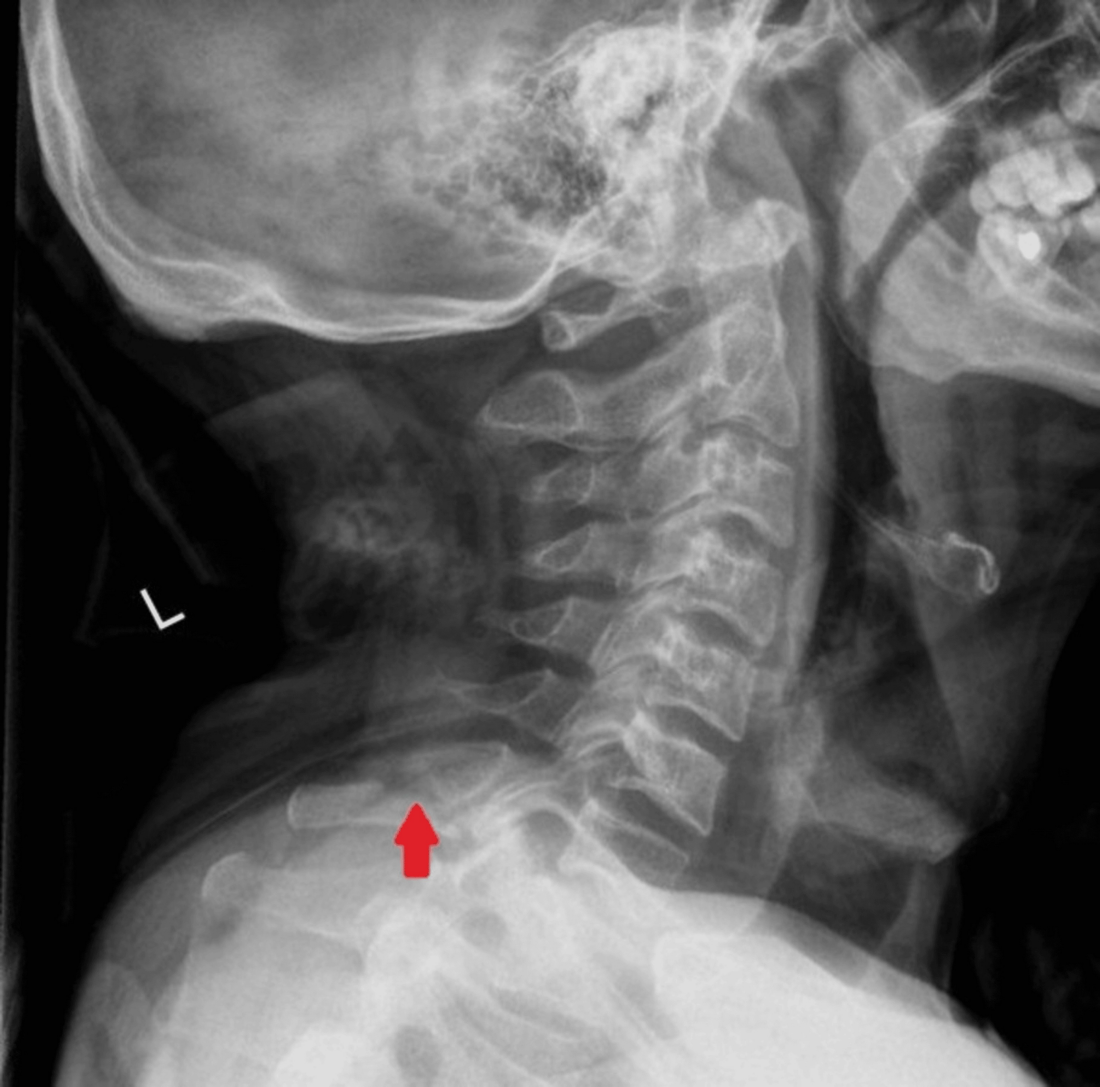
Lateral view cervical spine X-ray showing the previously identified C7 spinous process fracture (red arrow).

MRI of the cervical spine was performed the next day to rule out any spinal cord and ligament injury, confirmed the presence of a C7 spinous process fracture, and revealed an interspinous ligament tear immediately below the fracture, with edema of the interspinous ligament from the C2 to T1 vertebral levels (Figure 2). Other ligamentous structures, including the anterior and posterior longitudinal ligaments, ligamentum flavum, supraspinous ligament, and ligamentum nuchae, were intact. The spinal cord appeared normal. The patient was discharged on the fifth day of admission. A cervical spine X-ray was performed before discharge, and it reported no new findings except for the previously identified C7 spinous process fracture and no gross displacement of the fractured fragment (Figure 3). He was prescribed analgesia and instructed to continue collar use, with outpatient follow-up scheduled in two weeks.

**Figure 4:**
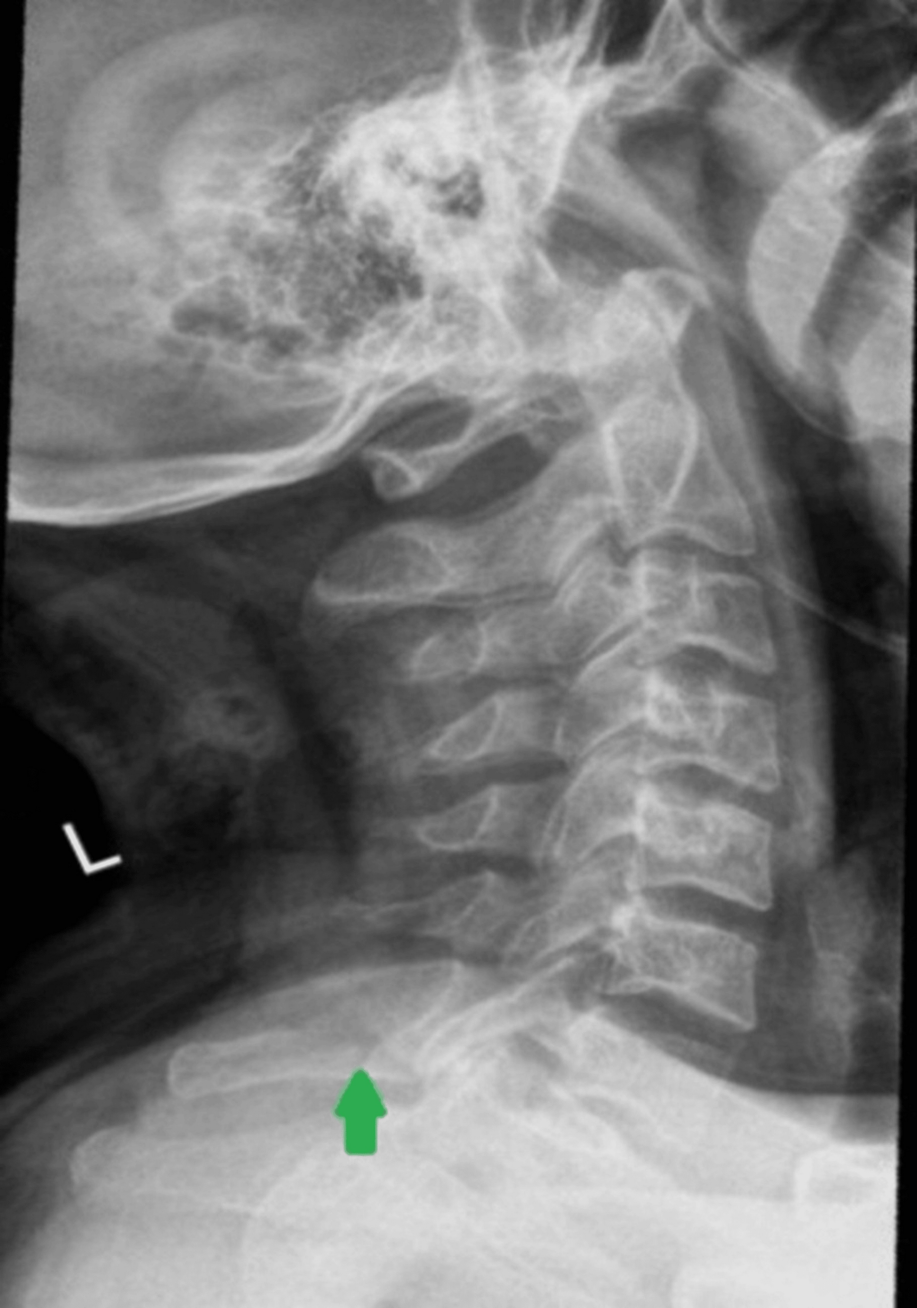
OPD follow-up cervical spine X-ray (lateral view) demonstrating the previously diagnosed C7 spinous process fracture without any interval change in the alignment or appearance of the fracture. OPD: outpatient department

At follow-up in the outpatient clinic, he reported ongoing neck pain. A repeat cervical spine X-ray was obtained, which was also noted as unremarkable for any new acute abnormality by both the ordering clinician and the reporting radiologist, except for the previously diagnosed C7 spinous process fracture (Figure 4). Collar use was extended for an additional four weeks. Two weeks later, he re-presented to the emergency department with persistent neck pain and new right shoulder discomfort. Examination revealed cervical tenderness from C1 to T1 and localized anterior tenderness of the right shoulder with mild limitation in abduction. Neurological and limb exams were normal. A right shoulder X-ray was obtained, which was normal, and the patient was discharged with analgesics and advised physiotherapy for right shoulder pain.

**Figure 5:**
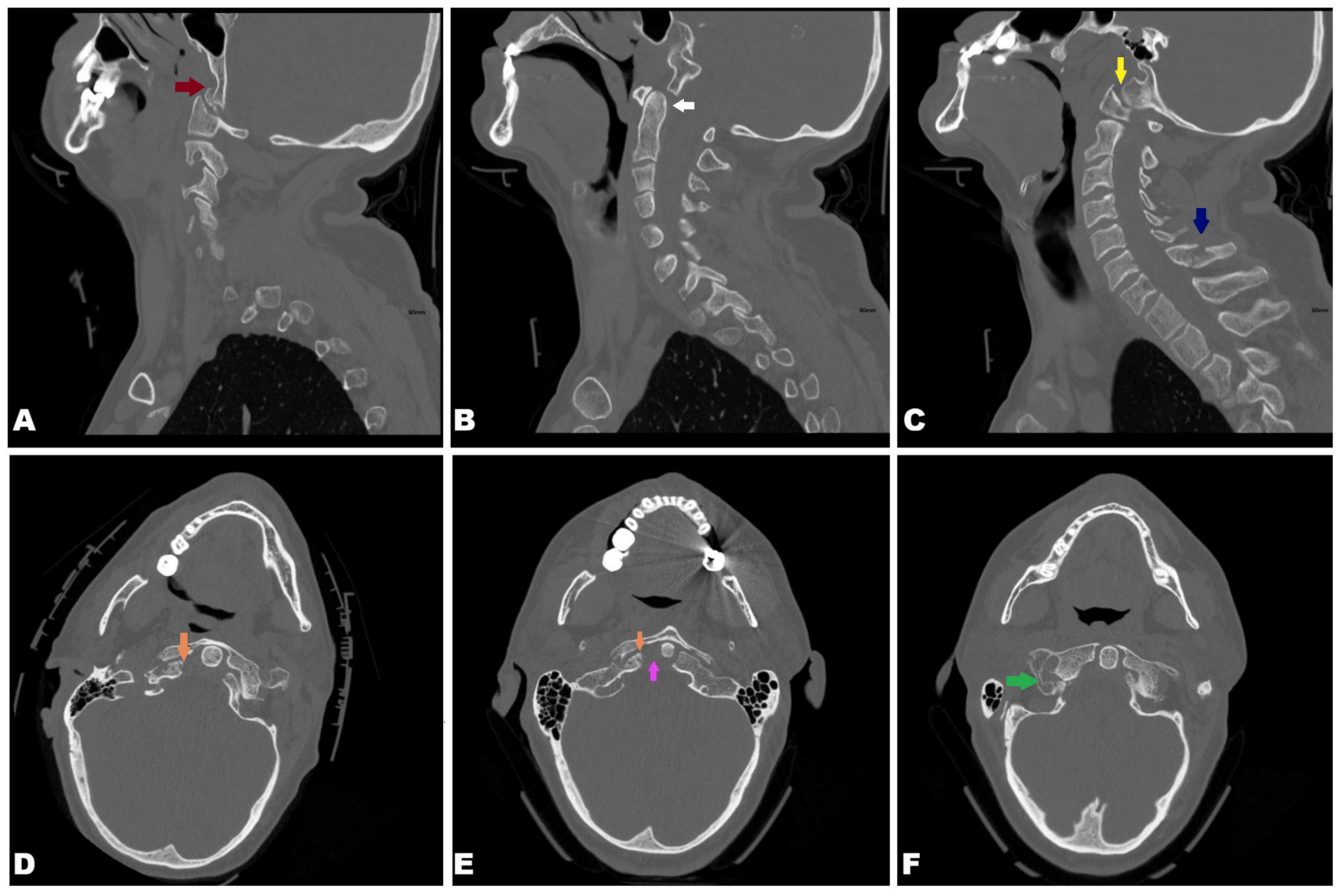
Sagittal (A, B, C) and axial (D, E, F) view CT images of the cervical spine demonstrating C7 fracture (subfigure C, blue arrow) and multiple previously missed injuries. Findings include a comminuted right occipital condyle fracture (subfigure D and E, orange arrows), a right C1 lateral mass fracture extending into the right foramen transversarium (subfigure F, green arrow) with associated impaction on the right occipital condyle (subfigure A, red arrow), bilateral widening of the atlanto-occipital joint spaces (subfigure C, yellow arrow), widening of right odontoid lateral mass interspace (subfigure E, pink arrow) and atlanto-occipital subluxation (subfigure B, white arrow). CT: computed tomography

Two days later, a different radiologist, while reporting the recent shoulder radiograph, retrospectively reviewed the prior cervical spine CT imaging and identified the previously missed findings: a comminuted right occipital condyle fracture, a right C1 lateral mass fracture extending into the foramen transversarium, impaction of the right C1 lateral mass on the occipital condyle, bilateral widening of the atlanto-occipital joint spaces, and subtle atlanto-occipital subluxation. An undisplaced right transverse process fracture of C1 involving the foramen transversarium was also present (Figure 5).

**Figure 6:**
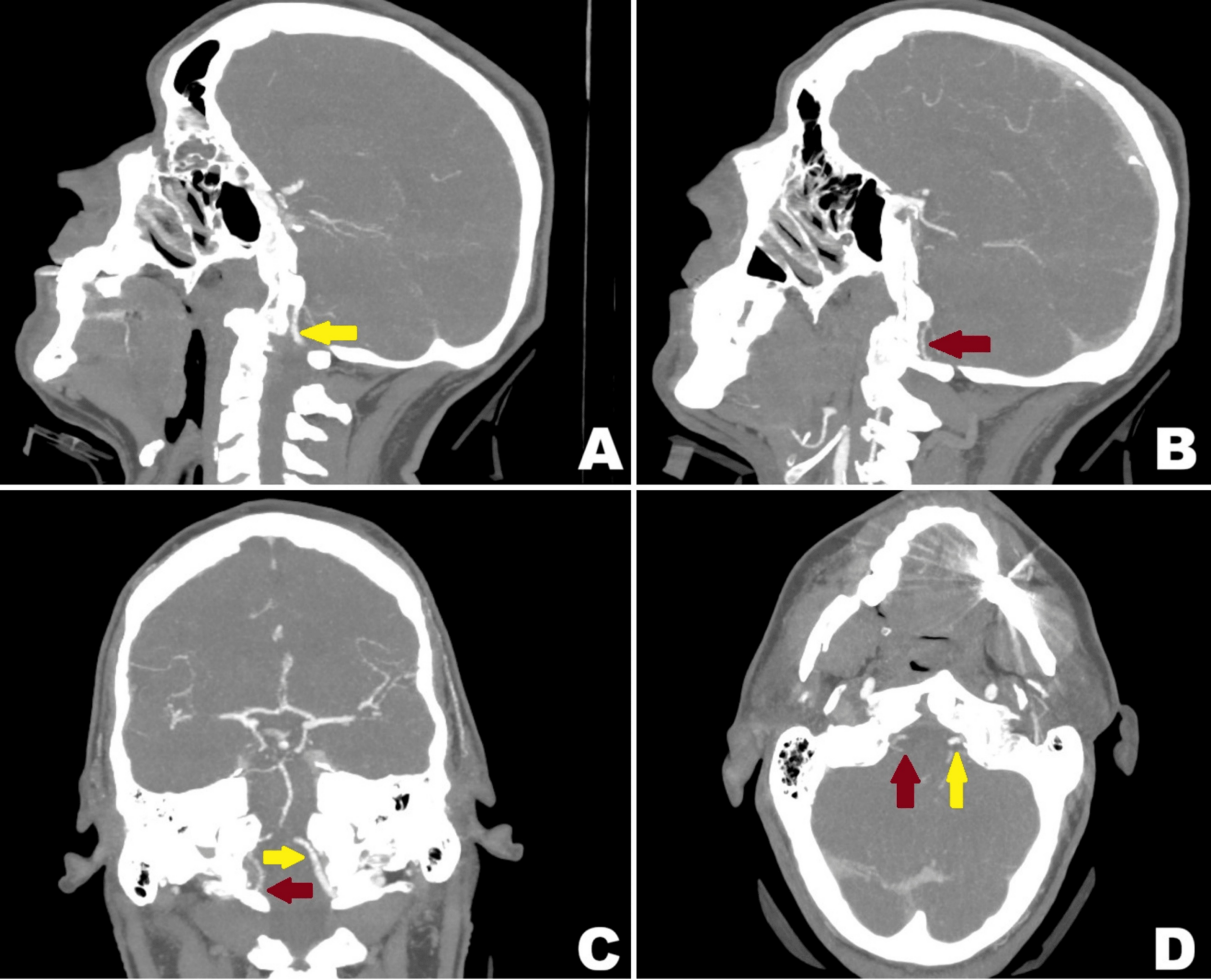
CT angiogram (vascular window) of the head and neck (A, B, C, D) showing subtle mural irregularity of the right vertebral artery (subfigure C, red arrow) at the level of the C1 fracture site. The right V4 segment appears diminutive (subfigure B, C and D, red arrows) compared to the left (subfigure A, C and D, yellow arrow), findings suggestive of a possible intimal injury. CT: computed tomography

The patient was immediately recalled to the emergency department for urgent assessment. Repeat CT of the cervical spine confirmed these findings and redemonstrated the C7 spinous process fracture with bilateral extension into the lamina, without significant callus formation. CT angiography was also performed to rule out vertebral artery injury, revealing subtle mural irregularity of the right vertebral artery at the fracture site and a diminutive V4 segment, suggesting possible intimal injury (Figure 6). The basilar artery and left vertebral artery were patent.

Repeat MRI demonstrated edema of the right occipital condyle extending to the inferior clivus, mild marrow edema in the right C1 lateral mass, widening of the atlanto-occipital joints, and impaction of the right C1 lateral mass on the occipital condyle. The anterior and posterior atlanto-occipital membranes, transverse ligament, anterior and posterior longitudinal ligaments, ligamentum flavum, ligamentum nuchae, and supraspinous ligament were intact. The apical ligament was poorly visualized, and injury could not be excluded. The spinal cord remained normal.

The patient was referred to a neurosurgical center for further management, but did not attend follow-up appointments despite multiple attempts and was ultimately lost to follow-up.

## Discussion

This case highlights multiple radiologic interpretation challenges that contributed to the delayed recognition of significant craniocervical injuries. While appropriate imaging, including CT, X-ray, and MRI, was performed, key injuries were missed due to cognitive biases and an incomplete systematic review of the craniovertebral junction.

Fractures of the occipital condyle and C1 lateral mass are often subtle and can be overlooked, particularly when more obvious injuries, such as a C7 spinous process fracture, are present [1,2]. In this case, the initial obvious C7 fracture likely triggered anchoring bias, whereby subsequent interpretation was disproportionately influenced by this finding. Satisfaction of search also contributed, as the detection of one abnormality led to premature termination of image review. Additionally, adherence bias may have played a role, as treating clinicians and subsequent radiologists appeared to rely on previous reports and did not systematically re-evaluate imaging despite the patient's persistent clinical symptoms. Together, these biases resulted in the occipital condyles, C1 ring, atlanto-occipital joints, and foramina transversaria being inadequately assessed for the missed injuries.

In addition to cognitive factors, contextual and system-level contributors may have influenced the missed diagnosis. The injuries were identified only when a different radiologist later reviewed the prior imaging while reporting an unrelated shoulder radiograph. Had the same radiologist been responsible for both studies, the prior CT may not have been re-examined in detail, and the injuries could have remained unrecognized. Differences in radiologist experience, workload levels, reporting environment, or cognitive fatigue at the initial interpretation may also have affected diagnostic accuracy. These elements should be considered as contributing factors to the interpretive error.

A systematic approach to cervical spine trauma imaging could have mitigated these errors [3]. Careful assessment of alignment, joint spaces, osseous continuity, and foraminal integrity is critical while reporting spine imaging. Retrospective review confirmed that fractures of the right occipital condyle and right C1 lateral mass, as well as subtle atlanto-occipital subluxation (Traynelis type III), were visible on the initial CT [7]. Early MRI also failed to highlight these injuries, as the reporting radiologist focused primarily on the C7 fracture and an expected interspinous ligament tear, and did not fully evaluate ligamentous integrity at the craniovertebral junction.

Missed recognition of the foramen transversarium involvement delayed assessment of the vertebral artery. When CT angiography was finally performed, a subtle mural irregularity of the right vertebral artery was noted, illustrating the potential risk of vascular injury associated with missed upper cervical fractures.

The injuries were ultimately identified only when a different radiologist retrospectively reviewed the prior imaging during reporting of an unrelated shoulder radiograph, underscoring the importance of second-reader review, re-evaluation of prior imaging in symptomatic patients, and vigilance against cognitive biases [8]. Structured reporting protocols, systematic search patterns, and correlation with persistent clinical findings are essential to prevent missed injuries [9].

Despite the delayed diagnosis, the patient did not experience neurological deterioration, likely due to continuous cervical immobilization. Nonetheless, the case emphasizes that undetected craniocervical injuries can have serious consequences and highlights the need for radiologists to maintain a high level of vigilance for subtle injuries and cognitive biases.

## Conclusions

This case demonstrates delayed recognition of a right occipital condyle fracture, C1 lateral mass fracture extending into the foramen transversarium, and subtle atlanto-occipital subluxation, all initially missed on CT, MRI, and follow-up radiographs. Cognitive biases - including anchoring, satisfaction-of-search, and adherence bias - played a central role in these oversights. Persistent symptoms, retrospective review, and evaluation by a different radiologist were crucial to the eventual diagnosis.

Radiologists must adopt and follow systematic approaches to imaging review, carefully assess subtle and high-risk anatomical regions, and reconsider prior studies when patients continue to experience symptoms. Awareness of cognitive biases, particularly adherence bias, is essential to prevent over-reliance on previous reports and to ensure early detection of injuries that could otherwise result in neurological or vascular complications. This case highlights the importance of structured reporting, clinical correlation, and multidisciplinary discussion in trauma imaging.
